# The association of dorsal and ventral white matter tracts with phonological and semantic processing of language in 5- to 7-year-old children

**DOI:** 10.1016/j.dcn.2025.101662

**Published:** 2025-12-19

**Authors:** Avantika Mathur, Huijia Zheng, Yingying Wang, Marjolein Mues, James R. Booth

**Affiliations:** a Brain Development Laboratory, Department of Psychology and Human Development, Peabody College, Vanderbilt University, Nashville, TN 37203, United States; b NL3 lab, Department of Special Education and Communication Disorders, University of Nebraska–Lincoln, Lincoln, NE 68583-0738, United States

**Keywords:** Phonology, Semantics, Diffusion tensor imaging, Specialization, Children, Arcuate Fasciculus, Inferior fronto-occipital fasciculus

## Abstract

This preregistered Diffusion Tensor Imaging (DTI) study aims to investigate the functional dissociation between left dorsal and ventral white matter tracts during language development. We examine the unique relations of dorsal tracts, including the Superior Longitudinal Fasciculus, Arcuate Fasciculus, to phonological processing and ventral tracts, including the Inferior Longitudinal Fasciculus and Inferior Fronto-Occipital Fasciculus, to semantic processing. Automatic Fiber Quantification was performed on DTI scans of 81 5-year-olds and 164 7-year-olds to map and segment white matter tracts. Partial correlation analysis was conducted to assess the relationship between language skill and white matter integrity (measured via fractional anisotropy) of tracts at the node-level. In 5-year-olds, the results revealed that the tract integrity of the dorsal Arcuate Fasciculus tract (nodes 68–87) showed a unique positive relation with a standardized measure of phonological processing (i.e. Elision). In 7-year-olds, the tract integrity of the ventral Inferior Fronto-Occipital Fasciculus (nodes 41–57) showed a unique positive relation with a standardized measure of semantic processing (i.e. Word Classes). These findings suggest the importance of phonological processing during early language development (age 5) while during later stages (age 7), semantic mechanisms become more prominent.

## Highlights

• Phonological processing is linked to the integrity of a dorsal white matter tract in early language development.
• Semantic processing is related to ventral white matter tract integrity in later language development.
• The Arcuate Fasciculus exhibits a unique relationship with phonological skills in 5-year-olds.
• The Inferior Fronto-Occipital Fasciculus demonstrates a unique correlation with semantic skills in 7-year-olds.

## 1 Introduction

According to the triangle model of reading, reading is achieved through a mechanism where inputs from orthographic, phonological, and semantic pathways interact to produce an output (Seidenberg, 2005). Specifically, two pathways work together to achieve phonological output (i.e., reading aloud): one where orthography connects to phonology via hidden units representing smaller elements like letters and phonemes, and another where orthographic input activates semantic representations that, in turn, activate phonology. Efficient reading results from combining inputs from both pathways, with the division of labor between them varying based on the word type (Harm and Seidenberg, 1999, Harm and Seidenberg, 2004). These pathways are interdependent, meaning changes in one affect the entire system. The foundational step in learning to read involves recognizing that spoken words can be broken down into basic sound units, known as phonemes, and connecting these to a limited set of written characters, or graphemes (Harm and Seidenberg, 1999). Beginners in reading primarily decode grapheme-phoneme correspondences to map between the spelling of a word, i.e., orthography, and its pronunciation, i.e., phonology. As this decoding reading proficiency grows, children transition to effectively map spellings to meanings, i.e. semantics (Pugh et al., 2001, Schlaggar and McCandliss, 2007). The research seems to converge on the idea that early in reading decoding from orthography to phonology mapping dominates, and later in reading children acquire more robust mapping from orthography to semantics (Ehri, 2008, Harm and Seidenberg, 2004, Vellutino et al., 2007, Ziegler and Goswami, 2005).

Researchers have employed diffusion tensor imaging (DTI) to study the white matter connections between brain regions involved in reading related processes. White matter connections are bundles of myelinated axons that facilitate neuronal communication by allowing signals to be transmitted rapidly between different brain regions. DTI measures the degree and directionality of water diffusion within brain voxels. White matter voxels exhibit a high degree of diffusivity parallel to the tract direction, as water can more easily diffuse along myelinated axons than perpendicular to them. Fractional anisotropy (FA) values reflect this directional diffusivity (Basser et al., 1994, Basser, 1995). Higher FA values are suggested to be associated with increased integrity and structural coherence within a tract (Mori, 2007), which may indicate more efficient information processing along the tract and greater connectivity between the endpoint brain regions. The goal of our project was to determine whether certain white matter tracts were uniquely related to behavioral measures of phonological processing and others were uniquely related to semantic processing, measured using oral language tasks in 5- and 7-year old children. We were also interested in investigating whether these effects were overlapping with correlations to reading skill.

**Fig. 1:**
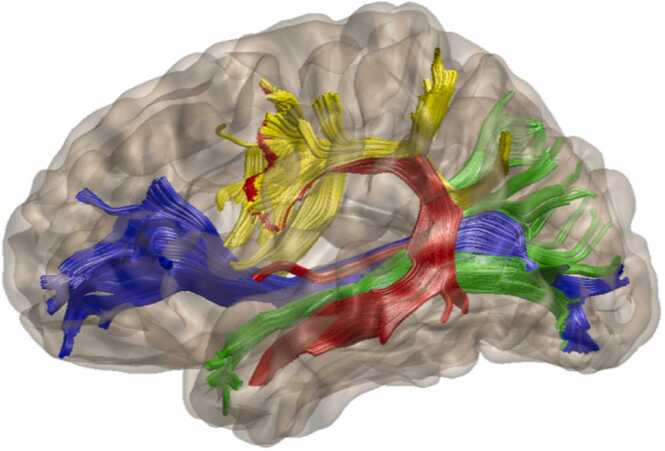
*Four white matter tracts of interest. Note.* The four major tracts IFOF (blue), ILF (green), AF (red), SLF (yellow) are represented using the AFQ software for one participant from the dataset analyzed in this study.

In adults, white matter tracts that support language processing have been well studied and are classified into dorsal and ventral streams (Friederici, 2015, Shekari and Nozari, 2023). While the triangle model does not directly represent neural mechanisms, the dorsal stream's information flow parallels the phonological pathway, and the ventral stream parallels the semantic pathway. The phonological pathway is suggested to be sustained by left dorsal regions connected by the Superior Longitudinal Fasciculus (SLF) (Kyeong et al., 2019, McKinnon et al., 2018) and Arcuate Fasciculus (AF) (Jarret et al., 2022, Schwartz et al., 2012, Yamada et al., 2007). The SLF connects the temporoparietal area with the inferior frontal gyrus, and the AF connects posterior superior temporal cortex to the inferior frontal gyrus and ventral premotor cortex. On the other hand, the semantic pathway is suggested to be sustained by the left Inferior Fronto-Occipital Fasciculus (IFOF) (Mirman et al., 2015) and the left Inferior Longitudinal Fasciculus (ILF) (Harvey and Schnur, 2015). The IFOF is a large multilayered tract running through the extreme and external capsules and connecting occipitotemporal and parietal regions with inferior frontal gyrus and the dorsolateral prefrontal cortex, and the ILF connects the occipital cortex with anterior temporal lobe, see Shekari and Nozari, (2023), for an overview of these white matter tracts, and refer to Fig. 1. However, the developmental specificity of white matter tracts of dorsal and ventral streams for phonological and semantic processing remains unclear.

In children across a wide age range, the correlation between tract integrity of the dorsal and ventral white matter tracts with reading ability has often been demonstrated using Letter-Word Identification as a measure of reading skill, where children are asked to read aloud printed words of varying lengths. Positive correlations have been shown between Letter-Word Identification skills and tract integrity (FA) of the dorsal tracts including AF (Gullick and Booth, 2014, Gullick and Booth, 2015, Yeatman et al., 2011) and SLF (Borchers et al., 2019, Bruckert et al., 2019, Travis et al., 2017), as well as the ventral tracts including ILF (Broce et al., 2019, Horowitz-Kraus et al., 2014; Y. Wang et al., 2017) and IFOF (Horowitz-Kraus et al., 2014, Vanderauwera et al., 2018, Vandermosten et al., 2015).

In older children, there is some evidence of a double dissociation of function in the dorsal and ventral white matter tracts (Cross et al., 2023, Su et al., 2018). Su et al. (2018) reported a functional dissociation between the left dorsal AF and the ventral ILF pathways in Chinese dyslexic children (average age of 11 years). FA values along the left AF correlated uniquely with phonological processing (assessed using a composite score obtained from phoneme deletion, rapid automatized naming, digit recall), while FA values along the left ILF were uniquely correlated with morphological processing skill (assessed with a task that required producing new words with a target morpheme). This dissociation supports the notion of unique roles for these pathways. In addition, Cross et al. (2023) examined children aged 8–14, finding that higher FA in the left AF correlated positively with oral reading of single words and rapid naming abilities. These effects persisted when controlling for other reading subskills, suggesting the left AF plays a specific role in oral reading and rapid naming. In contrast, higher FA in the right ILF was correlated with lower reading comprehension. These negative associations may reflect compensation in weaker readers. They argued for a dissociation between dorsal and ventral tracts, with the right ILF being involved for reading comprehension, which is heavily reliant on semantic representations, and the left AF being involved in phonological processing.

There is consistent evidence pointing to the role of the dorsal left AF in phonological processing, observed both before children start to learn to read (i.e. 5–6 year olds) (Saygin et al., 2013, Vandermosten et al., 2015; Y. Wang et al., 2017) and as they become more proficient readers (i.e., 7–11 year olds) (Yeatman et al., 2011). This tract is linked to the ability to map letters to sounds, or grapheme-phoneme conversion (Gullick and Booth, 2014). For the dorsal left SLF, there are contradictory results from studies. Some have supported the function of left SLF in phonological processing before children start learning to read. For example, Travis et al. (2017) have shown that FA in the anterior segment of left SLF is significantly correlated with phonological awareness in 6-year-olds. However, Saygin et al. (2013) found no correlation between phonological awareness and white matter integrity of the left SLF in children between 5 and 6 years of age. Thus, while there is evidence pointing to the specialization of the left AF in phonological processing, the left SLF does not appear to exhibit such specialization.

The role of the ventral tracts in early reading related processes, particularly the left IFOF and left ILF, has conflicting findings. Vandermosten et al. (2015) posited that the left IFOF is engaged in phonological processing in children ages of 5–6, but Vanderauwera et al. (2018) contended that the left IFOF is implicated in accessing meaning from orthography from the outset of reading acquisition. In a longitudinal study, they examined FA in ventral pathways in 5–6-year-olds and correlated the tract integrity with phonological processing and orthographic knowledge after two years of reading acquisition (7–8 years old). They found that, after two years of reading experience, the structural integrity of the left IFOF was correlated with orthographic knowledge, but not with phonological processing (Vanderauwera et al., 2018). These findings suggest that the ventral white matter tracts contribution to early reading is particularly pronounced for orthographic processing. They also reported that the specific role of the left ILF in 5–6-year-olds was ambiguous, with no clear link to either orthographic or phonological processing. Adding to the contradictory results, a study by Broce et al. (2019) shows that the left ILF is predictive of phonological awareness in children aged 5–8, indicating a role typically ascribed to the dorsal pathways. Thus, while there is evidence pointing to the specialization of the left IFOF in semantic aspects of reading in 7–8 years old, the left ILF does not appear to exhibit such specialization.

The discrepancy across studies may be attributed to developmental changes in the importance of different language functions to reading, with some studies suggesting that during reading acquisition, children undergo a gradual shift from an emphasis on phonological to semantic processing (Yeatman et al., 2012). However, there is no conclusive DTI evidence for differential association of reading related skills to dorsal versus ventral tracts across development.

The present study aims to investigate whether there is a double dissociation between the functions of dorsal and ventral white matter tracts in 5-year-olds and 7-year-olds. We expect that there would be strong evidence for phonological specialization in the dorsal tracts in 5-year-olds and strong evidence for semantic specialization in the ventral tracts in 7-year-olds. Specifically, we hypothesized that the left AF would be uniquely related to phonological processing in 5-year-olds, while the left IFOF would be uniquely related to semantic processing in 7-year-olds. For the left SLF and left ILF, no firm hypothesis was formulated for either group, given their inconclusive role in phonological or semantic processing (Broce et al., 2019, Saygin et al., 2013, Vanderauwera et al., 2018). Understanding double dissociation would provide stronger support for developmental models of language that argue for a dorsal stream involved in phonology and a ventral stream in semantics (Friederici, 2012, Hickok and Poeppel, 2004, Saur et al., 2008). Double dissociation has been used in neuroscience to argue for independence of processing paths in the brain (Lomber and Malhotra, 2008). Our research aims to clarify previous work that has examined either phonological processing (Saygin et al., 2013, Vandermosten et al., 2015; Y. Wang et al., 2017) (Yeatman et al., 2011) or semantic processing (Vanderauwera et al., 2018, Vandermosten et al., 2015), without controlling for the other, limiting the ability to make claims about the specificity of functions of dorsal and ventral pathways.

## 2 Methods

### 2.1 Participants

From a total of 322 participants from the publicly available longitudinal dataset (J. Wang et al., 2022), 87 participants from the 5-year-olds and 191 participants from the 7-year-olds had DTI imaging data acquired. This dataset recruited participants from the Austin, Texas metropolitan area via online advertisements (e.g., Facebook, lab webpage ads), community fliers, brochures sent to schools and clinics and mailings distributed to parents. These participants were then filtered based on quality control criteria. Participants included in analysis had obtained at least a standard score of 70 on Kaufman Brief Intelligence Test (KBIT-2, Kaufman and Kaufman, 2004); had obtained a standard score of at least 80 on the Core Language Score from Clinical Evaluation of Language Fundamentals, 5th edition, (CELF-5, Semel et al., 2013); were Mainstream English Speakers, according to the Diagnostic Evaluation of Language Variation (DELV) test (Seymour et al., 2022); had a score within 3 standard deviations of the mean on the other ability tests of interest (Elision, Word Classes, Letter-Word Identification); had no clinical diagnosis of neurological, psychiatric or developmental disorders as reported in a parent questionnaire; and were right-handed, reported as completing at least 3 of 5 tasks (writing, drawing, picking-up, opening, and throwing) with the right hand.

After filtering based on quality control criteria, demographic analysis was performed upon the remaining 81 participants (51 Female, 30 Male, mean age = 5.73, SD = 0.26) from the 5-year-olds and 164 participants (93 Female, 71 Male, mean age = 7.41, SD = 0.33) from the 7-year-olds. In the USA, children generally receive formal reading instruction from the first grade. Children aged 5 were generally in kindergarten. 8 in pre-kindergarten, 60 were in kindergarten, 7 were in 1st grade, 1 was homeschooled and 5 did not report the grade. Children aged 7 were generally in first or second grade. 36 were in 1st grade, 39 were in 2nd grade, 3 were in 3rd grade and 86 did not report the grade. Given that maternal education is one of the strongest indicators of SES (Hoff et al., 2012), parents were asked to indicate their highest grade/degree with 1 corresponding to “high school,” 2 to “some college,” 3 to “associate degree,” 4 to “bachelor’s degree,” and 5 to “master’s degree or higher". A Wilcoxon rank-sum test was conducted to compare the levels of mother education between 5-year-olds and 7-year-olds. There was no significant difference in maternal education scores between 5-year-olds (median = 4, IQR = 1.35) and 7-year-olds (median = 4, IQR = 2); W = 6490.5, p = .475 (refer to Table 1).

**Table 1:** Demographic details of participants.

	**5-year-olds (n = 81)**	**7-year-olds (n = 164)**
Gender	51 Female, 30 Male	93 Female, 71 Male
SES (Maternal Education)	8 High school, 9 Some college, 3 Associate Degree, 35 Bachelor’s Degree, 25 Master’s Degree or Higher, 1 n/a	11 High school, 25 Some college, 8 Associate Degree, 70 Bachelor’s Degree, 40 Master’s Degree or Higher, 10 n/a
Grade Levels	8 in pre-kindergarten, 60 in kindergarten, 7 in 1st grade, 1 homeschooled and 5 did not report	36 in 1st grade, 39 in 2nd grade, 3 in 3rd grade and 86 did not report
Race/Ethnicity	69 white, 12 other (1 - Asian, 4 - Black or African American, 7 – More than one race)	134 white, 26 other (1 - American Indian or Alaskan Native, 2 - Asian, 6 - Black or African American, 17 – More than one race), 4 did not report

.

### 2.2 Behavioral measures

In addition to an MRI scan, participants were tested on several behavioral measures. Nonverbal intelligence (NVIQ) was assessed using the KBIT-2 (Kaufman and Kaufman, 2004). To confirm that the participant attained at least average language scores, the Core Language scale from the CELF-5 (Semel et al., 2013) was used. Phonological processing was evaluated with the Elision subtest from the Comprehensive Test of Phonological Processing, 2nd Edition (CTOPP-2; Wagner et al., 2013). This subtest asks participants to remove a phoneme from a spoken word to form a new spoken word, for instance, saying “tiger” without the “g” would result in “tire”. Semantic processing was assessed with the Word Classes subtest (CELF-5; Semel et al., 2013). This subtest instructs participants to choose two words with a semantic relationship from three or four presented words, for instance, choose “puppy” and “dog” when presented with “puppy, dog, frog”. Reading skill was measured with the Letter-Word Identification subtest (WJ-III; (Woodcock et al., 2001), which asks participants to orally read letters and words from a printed list. The average standardized scores with standard deviation and range for NVIQ (KBIT-2), Core Language (CELF-5), and Letter-Word Identification (WJ-III) are indicated in Table 2. The average scaled Scores with standard deviation and range for Elision (CTOPP-2) and Word Classes (CELF-5) are also indicated in Table 2.

**Table 2:** Standardized scores of participants.

	**5-year-olds (n = 81)**		**7-year-olds (n = 164)**		**Partial Overlap *t*-test**	
**Measure**	**Mean (SD)**	**Range**	**Mean (SD)**	**Range**	**t (df) =**	**p**
Age (years)	5.7 (0.3)	5.0–6.3	7.4 (0.3)	7.0–8.3		
NVIQ (KBIT-2)^b^	105.7 (15.0)	80–151	108.0 (17)	74–147	-1.04 (169.30)	= 0.30
Core Language (CELF-5)^b^	114.7 (12.1)	92–147	103.7 (14.0)	80–139	7.04 (169.90)	< 0.001
Elision (CTOPP-2)^a^	11.5 (2.1)	6–18	11.0 (2.8)	5–17	2.01 (142.79)	= 0.05
Word Classes (CELF-5)^a^	12.8 (3.3)	6–19	12.0 (3.3)	3–19	2.06 (169.90)	= 0.04
Letter-Word Identification (WJ III)^b^	122.5 (16.0)	94–164	115.5 (12.3)	83–144	4.37 (169.90)	< 0.001

Note. The average standardized scores with standard deviation and range for KBIT-2, Kaufman Brief Intelligence Test, CELF-5, Clinical Evaluation of Language Fundamentals, CTOPP-2, Comprehensive Test of Phonological Processing, WJ-III, Woodcock-Johnson III Tests of Achievement are indicated. The t-value (degree of freedom) and p-value for each Partial Overlap *t*-test between the scores of 5-year-olds and 7-year-olds cohort are also indicated.

a Scaled scores (population mean of 10, *SD* of ±3)

b Standard scores (population mean of 100, *SD* of ±15)

Partial Overlap *t*-tests (Derrick et al., 2017) were conducted in R using the function Partover.test to compare the standard scores across the 5-year-old cohort and 7-year-old cohort. Table 2 indicates the t-value (degrees of freedom) and p-value for each test. There were no differences between the cohorts on Non-Verbal Intelligence. In contrast, the 5-year-olds tended to score higher than the 7-year-olds on the language and reading measures, indicating a cohort effect. To be clear this analysis shows differences between the two cohorts relative to their peers. This indicates behavioral differences are smaller than would be expected based on population means, but it is still the case the raw scores of the 7-year-olds were higher than the 5-year-olds (see supplementary material). A partially overlapping samples *t*-test (equal-variance) showed that 5-year-olds (M = 12.83 (SD = 3.31, n = 81)) had higher scaled scores on Word Classes than 7-year-olds (M = 12.01 (SD = 3.30, n = 164)). A partially overlapping samples *t*-test (Welch-type) showed that 5-year-olds (M = 11.52 (SD = 2.14, n = 81)) had higher scaled scores on Elision than 7-year-olds (M = 10.97 (SD = 2.77, n = 164)). The means of scaled scores show that the differences were relatively modest, at less than one third of the standard deviation for these measures (Elision and Word Classes) used in the brain-behavior analysis to examine unique relations with phonology and semantics. In addition, partially overlapping samples *t*-tests (equal-variance) indicated that the 5-year-old cohort had significantly higher standard scores on Letter-Word Identification and Core Language than the 7-year-old cohort.

It is to note that the raw scores of Elision, Word Classes and Letter-Word Identification were used in statistical analysis (see statistical analysis section below).

## 3 Image acquisition

### 3.1 Structural MRI

T1-weighted Magnetization Prepared - RApid Gradient Echo (MPRAGE) images were collected using GRAPPA, a parallel imaging technique based on k-space, and the following parameters: GRAPPA accel.factor PE = 2, TR = 1900 ms, TE = 2.43 ms, field of view = 256 mm, matrix size = 256 × 256, band-width = 180 Hz/Px, slice thickness = 1 mm, number of slices = 192, voxel size = 1 mm isotropic, flip angle = 9°.

### 3.2 Diffusion weighted imaging

Diffusion-weighted images were collected using single-shot echo planar imaging (EPI) with the following parameters: GRAPPA accel.factor PE = 2, TR = 5000 ms, TE = 71 ms, field of view = 256 mm, matrix size = 128 × 128, bandwidth = 1502 Hz/Px, slice thickness = 2 mm, number of slices = 57, voxel size = 2 mm isotropic, flip angle = 90°, multiband acceleration factor = 3, b-value1 = 0 s/mm2, b-value2 = 800 s/mm2, echo spacing = 0.75 ms, diff.directions = 64. Slices were acquired interleaved from foot-to-head.

### 3.3 DTI quality control

The T1-weighted structural image was used to generate a brain mask by removal of non-brain tissue using the Brain Extraction Tool (BET) (Smith, 2002) from functional MRI of the Brain (FMRIB) software Library (Oxford, UK). DWI DICOM data was converted into NRRD (teem.sourceforge.net/nrrd/) format using DicomToNrrdConverter software from Slicer4 (www.slicer.org). DWI quality control (QC) procedures were conducted using DTIprep software (Oguz et al., 2014) and visual inspection. Motion artifacts were defined by translation threshold of 2.67 mm and rotation threshold of 0.5° through rigid registration-based volume-by-volume measures. Volumes with motion artifacts were excluded from diffusion tensor estimation (a volume was removed if it contained 3 or more bad slices). After QC, DWI data was processed using mrDiffusion, a toolbox from the VISTALab (Stanford Vision and Imaging Science and Technology) diffusion MRI software suite (www.vistalab.com) including Eddy current correction and tensor-fitting estimations (Rohde et al., 2004). Diffusion tensors were fitted using a linear least-squares (LS) fit, and eigenvalues from the diffusion tensor estimation were used to compute FA (Basser et al., 1994).

### 3.4 Automatic fiber quantification

The AFQ (github.com/jyeatman/AFQ) software package (Yeatman et al., 2012) was used to identify white matter tracts. The AFQ analysis pipeline is described in greater detail in (Yeatman et al., 2012). AFQ uses a three-step procedure to identify 20 major fiber tracts in an individual's brain. The procedure is based on a combination of the methods described by Hua et al. (2008) and Zhang et al. (2008): (1) fiber tractography using a deterministic streamlines algorithm (STT) (Basser et al., 2000, Mori et al., 1999), (2) waypoint region-of-interest (ROI)-based fiber tract segmentation described in Wakana et al. (2007) and (3) fiber tract refinement based on a probabilistic fiber tract atlas (Hua et al., 2008). We used the AFQ_create function set with its default parameters to perform tractography on individual subjects (https://github.com/jyeatman/AFQ/blob/master/functions/AFQ_Create.m). The codes used in analysis are shared on GitHub (see the section data and code availability).

Each fiber tract was sampled at 100 equidistant nodes, at each node (certain spatial location) FA values were calculated. AFQ segments the whole-brain fiber group into 20 white matter tracts defined by an atlas (Wakana et al., 2007). The analyses here were focused on 4 left-hemispheric tracts: AF, SLF, IFOF and ILF (Fig. 1). Moreover, instead of computing mean diffusion parameters, AFQ computes diffusion parameters using a weighted sum of each fiber’s value at a given node where a fiber is weighted based on its Mahalanobis distance from the core or mean location of the tract (Johnson et al., 2014). This improves detection power for group differences. For one participant from the 7-year-olds the tractography using AFQ did not complete, thus this participant was removed from subsequent statistical analysis.

### 3.5 Statistical analysis

We performed AFQ analysis upon the white matter tracts of interest (SLF, AF, ILF and IFOF) which divides each tract into 100 equidistant nodes, and computed correlations at the node level. This analysis was done for both 5-year-olds and 7-year-olds (for tract profiles from AFQ, see supplementary figures (S1, S2, S3 and S4). To find nodes within the dorsal tracts (left AF and left SLF) that were uniquely related to phonological processing, we performed partial correlation analysis between Elision raw score and the FA value at each node along the tracts, controlling for Word Classes raw score, age and NVIQ. To find nodes within the ventral tracts (left IFOF and left ILF) that were uniquely related to semantic processing, we performed partial correlation analysis between Word Classes raw score and the FA value at each node along the tracts, controlling for Elision raw score, age and NVIQ. Finally, to find nodes within each tract that were related to reading ability, we performed partial correlation analysis between Letter-Word Identification raw score and the FA value at each node along all tracts of interest (AF, SLF, IFOF, and ILF), controlling for age and NVIQ. The age and NVIQ have been shown to be related to reading skill (Peng et al., 2019), thus were used as control variables in partial correlation analyses.

To control for multiple comparisons, we performed an implementation of the permutation method described by Nichols and Holmes (2002) and used in several studies (David et al., 2020, Yeatman et al., 2012). By modifying and running the function AFQ_MultiCompCorrection to correct partial correlation results, the data was randomly shuffled for 1000 permutations and a distribution of “chance” correlations for every correlation was created. We then created a final distribution from the maximum cluster size of these permutations, considering all the correlations, and compared the actual (nonshuffled) cluster size with these values to assign the significance alpha and cluster threshold at p < 0.05 (two-tailed). This returned a family-wise error (FWE) corrected cluster size, which means that significant clusters of this size or greater pass the multiple comparison threshold and do not need further p-value adjustment (Dodson et al., 2017, Dubner et al., 2020, Travis et al., 2015, Travis et al., 2016, Travis et al., 2017). Some previous studies have adopted a more lenient criteria (Banfi et al., 2019, Dodson et al., 2017), for example, with more than 3 of adjacent nodes under the quantification of the diffusion metrics along each fiber tract at 30 equidistant nodes (Dodson et al., 2017). Thus, in our study, planned comparisons were reported at a stringent threshold, requiring enough adjacent nodes to meet the criteria for a FWE corrected cluster size. Exploratory results were reported at a more lenient threshold, requiring ≥ 9 adjacent nodes along FA tract profiles resampled to 100 equally spaced nodes or ≥ 3 adjacent nodes along FA tract profiles resampled to 30 equally spaced nodes at p < 0.05 uncorrected. The analyses using 30-node profiles were conducted for confirmatory purposes, thus, the results obtained from resampling the FA tract profile to 30 equally spaced nodes at p < 0.05 uncorrected are reported in supplementary materials.

The introduction including the hypotheses, the statistical analytical plan including all the planned comparisons for both age 5 and age 7 conducted in this study were pre-registered (https://osf.io/dm78z).

In addition to the preregistered analyses, we conducted two more analyses. First, to make comparison with other published studies, we conducted partial correlations for each of the tracts and groups (age 5 and age 7) for phonological processing and semantic processing without controlling for the other process (only controlling for age and NVIQ). The results are incorporated in supplementary material.

Moreover, based on reviewer’ suggestions, we conducted partial correlations for each of the tracts and groups (age 5 and age 7) for phonological processing and semantic processing while controlling for the other process, age and NVIQ for only those participants with data at both time points. 45 participants were overlapping across the age 5 and age 7 groups after filtering based on quality control criteria. The results are incorporated in supplementary material.

## 4 Results

**Fig. 2:**
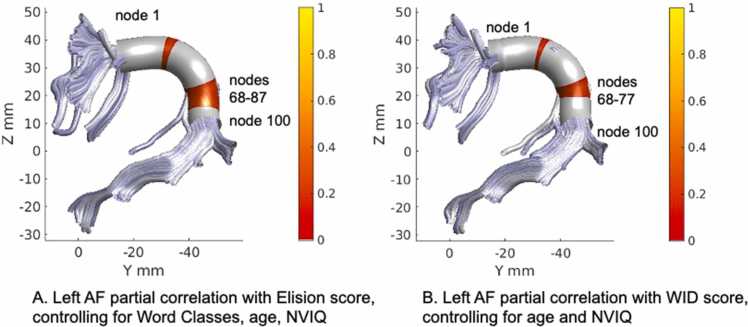
White matter partial correlations of fractional anisotropy (FA) in the left arcuate fasciculus (AF) with behavioral measures in 5-year-olds. Note. (A) Positive partial correlation with phonological processing (Elision), while controlling for semantics (Word Classes), age and nonverbal intelligence (NVIQ). (B) Positive partial correlation with Letter-Word Identification (WID) while controlling for age and nonverbal intelligence (NVIQ). The color bars show significant correlation values.

**Fig. 3:**
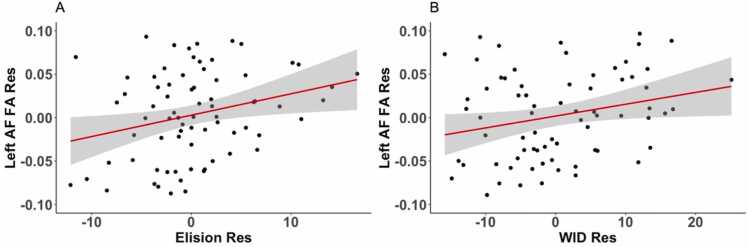
Scatterplots of FA in left arcuate fasciculus (AF) with behavioral measures in 5-year-olds. Note. Partial correlations are visualized as a scatter plot between residual FA values and residual values for each cognitive measure. The scatter plot (A) represents a positive correlation of mean FA of the left AF (nodes 68–87) with phonological processing (Elision), while controlling for semantics (Word Classes), age and nonverbal intelligence. The scatter plot (B) represents a positive correlation of mean FA of the left AF (nodes 68–77) with Letter-Word Identification (WID) while controlling for age and nonverbal intelligence.

In 5-year-olds, partial correlation analysis revealed a positive correlation between FA of left AF and the Elision score (nodes 34–39, nodes 68–87), the second cluster between nodes 68–87 survived the stringent threshold of FWE cluster size correction for adjacent nodes ≥ 15, at p < 0.05 uncorrected (Fig. 2A). A positive correlation was also observed within overlapping nodes between FA of left arcuate fasciculus and the Letter-Word Identification score (nodes 36–38, nodes 68–77) (the second cluster between nodes 68–77 survived the lenient threshold for adjacent nodes ≥ 9 at p < 0.05 uncorrected) (Fig. 2B). The moderate positive correlations are displayed in scatterplots for partial correlations (Figs. 3A and 3B), showing the residuals of Elision and Letter-Word Identification scores of age-5 participants plotted against residuals of mean FA of the left AF (nodes 68–87) (Fig. 3A) and mean FA of the left AF (nodes 68–77) (Fig. 3B). No significant correlations passing the cluster correction were observed between FA of SLF and the Elision score or the Letter-Word Identification score in 5-year-olds. No significant correlations passing the cluster correction were observed between FA of ventral tracts and the Word Classes score or the Letter-Word Identification score in 5-year-olds.

**Fig. 4:**
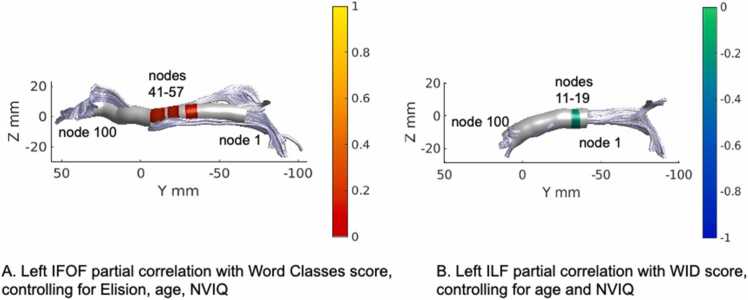
White matter partial correlations of fractional anisotropy (FA) in the left inferior fronto-occipital fasciculus (IFOF) and the left inferior longitudinal fasciculus (ILF) with behavioral measures in 7-year-olds. Note. (A) Positive partial correlation of the left inferior fronto-occipital fasciculus (IFOF) with semantics (Word Classes), while controlling for Elision, age and nonverbal intelligence (NVIQ). (B) Negative partial correlation of the left inferior longitudinal fasciculus (ILF) with Letter-Word Identification (WID) while controlling for age and nonverbal intelligence (NVIQ). The color bars show significant r-values.

**Fig. 5:**
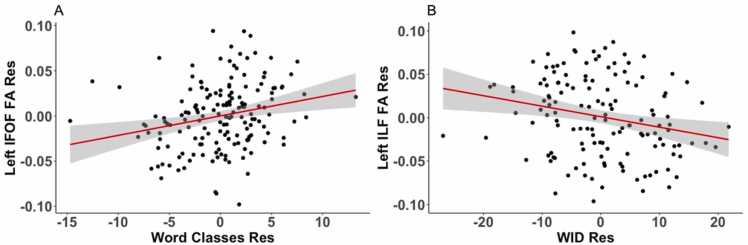
Scatterplots of FA in the left inferior fronto-occipital fasciculus (IFOF) and the left inferior longitudinal fasciculus (ILF) with behavioral measures in 7-year-olds. Note. Partial correlations are visualized as a scatter plot between residual FA values and residual values for each cognitive measure. The scatter plot (A) represents a positive correlation of the left inferior fronto-occipital fasciculus (IFOF) (nodes 41–57) with semantics (Word Classes), while controlling for Elision, age and nonverbal intelligence. The scatter plot (B) represents a negative correlation of the left inferior longitudinal fasciculus (ILF) (nodes 11–19) with Letter-Word Identification (WID) while controlling for age and nonverbal intelligence.

In 7-year-olds, partial correlation analysis revealed a positive correlation between FA of left IFOF and the Word Classes score (nodes 31–36, nodes 41–47, 50–57), the two clusters between nodes 41 and 57 did not survive the lenient threshold for adjacent nodes ≥ 9, but were close together with nodes 48 and 49 showing a correlation, r = 0.14, at p = 0.07 and r = 0.15, at p = 0.06 (Fig. 4A). The moderate positive correlation is displayed in scatterplot (Fig. 5A), showing the residuals of Word Classes scores of age-7 participants plotted against mean FA of the left IFOF (nodes 41–57). A negative correlation was also observed with FA of left ILF and the Letter-Word Identification score (nodes 11–19), at the lenient threshold for adjacent nodes ≥ 9 at p < 0.05 uncorrected (Fig. 4B). The moderate negative correlation is displayed in scatterplots for partial correlations (Fig. 5B), showing the residuals of Letter-Word Identification scores of age-7 participants plotted against mean FA of the left ILF. The FA of ILF showed no significant correlation with the Word Classes score. Otherwise, no significant correlations passing the cluster correction were observed between FA of dorsal tracts and the Elision score or the Letter-Word Identification score in 7-year-olds.

## 5 Discussion

The present study provides neurobiological evidence for a double dissociation between the functions of dorsal and ventral white matter tracts in 5-year-olds and 7-year-olds. Specifically, we hypothesized that the left AF would be uniquely related to phonological processing in 5-year-olds, while the left IFOF would be uniquely related to semantic processing in 7-year-olds. Based on the findings from previous studies, we did not have a firm hypothesis for the left SLF or the left ILF for either group given their inconclusive role in phonological or semantic processing (Saygin et al., 2013, Vanderauwera et al., 2018). In 5-year-olds, we found that the dorsal left AF tract shows a unique positive relation with phonological processing (measured using CTOPP-2 Elision subtest) while the ventral tracts (left ILF and left IFOF) did not show a unique relation with semantic processing (measured using CELF-5 Word Classes subtest). In 7-year-olds, the dorsal tracts (left AF and left SLF) did not show a unique relation with phonological processing while the ventral left IFOF showed a unique positive relation with semantic processing. These results suggest the importance of phonological processing during early language acquisition, as reflected by a positive correlation of white matter FA of the left AF with Elision at age 5. During later stages of development, semantic mechanisms seem to be more important in language acquisition, as evident from the positive correlation between white matter FA of the left IFOF and Word Classes at age 7. This pattern is consistent with dual-stream accounts in which dorsal pathways support phonology and ventral pathways support semantics (Hickok and Poeppel, 2007, Friederici, 2012). The relation of Elision with dorsal left AF in 5-year-olds overlapped with the association of this tract with reading skill as measured with Letter-Word Identification subtest, consistent with the triangle model of word reading (Seidenberg, 2005) by providing evidence for a greater reliance on phonological mechanisms during early reading acquisition. Together, our pre-registered analyses on the full sample suggest a developmental progression from early phonological involvement of the dorsal pathway at age 5 to increased ventral semantic contributions for language acquisition by age 7. Given limited overlap across age 5 and age 7 resulting in small sample sizes, future longitudinal studies need to confirm that these are not cohort effects.

### 5.1 Arcuate fasciculus (AF)

Consistent with our apriori hypotheses, the left AF was uniquely related to phonological manipulation in 5-year-olds, and this relation overlapped with reading skill, suggesting early reliance on phonology during decoding. In 7-year-olds, AF associations with phonology and reading were not observed, aligning with reports that AF–phonology links weaken with age. This developmental pattern accords with early-childhood studies that specifically implicate the left AF for phonological processing. Saygin et al. (2013) showed anatomically and behaviorally specific correlations between phonological awareness and FA in the left AF in 5–6-year-olds. There were no reported relations to rapid automatized naming or letter knowledge and no comparable effects in ILF or SLF. Similar to Saygin et al. (2013), Dodson et al. (2018) found that FA in the left AF contributed unique variance to phonological awareness in 6-year-olds (born pre-term or at-term) even after controlling for socio-economic status, sex, and nonverbal intelligence. In summary, in 5–6-year-olds, higher FA in the left arcuate fasciculus correlates with phonological awareness and overlaps with reading skill, indicating early, phonology-driven decoding. By contrast, in older samples the relation between phonology and white matter appear less robust. Consistent with our null finding of no correlation between AF and phonological awareness at age 7, Broce et al. (2019) reported no FA effect, although they did show a negative correlation for axial diffusivity in 7–11-year-olds. Our lack of correlations between AF and reading skill at age 7 differs from longitudinal evidence in broader, older cohorts showing that the AF predicts reading skill and exhibits differential age-dependent FA trajectories for good and poor readers (Yeatman et al., 2012, Gullick and Booth, 2015), possibly reflecting our narrower and slightly younger age range in age 7 cohort.

Our correlations between phonological awareness and AF in 5-year-olds was localized to posterior nodes, the portion with greater directional integrity compared to nodes in the middle where the corticospinal tract crosses and reduces FA (Yeatman et al., 2011; see Fig. S2). Posterior AF segments have been linked to behavior in other pediatric samples, including correlations with sight word reading and rapid naming (Cross et al., 2023) as well as with phonological awareness and reading skills (Gao et al., 2022), supporting a segment-specific account in which posterior AF is involved in phonological processing during early reading. Although AF is often linked to phonology, Broce et al. (2015) reported vocabulary skill in 5–8-year-olds was related to AF in the anterior segment. Our correlation between phonology and AF in 5-year-olds was posterior and specific to Elision, so in combination with previous literature, this suggests segment-specific, multifunctional AF contributions that vary with task demands and age span.

### 5.2 Superior longitudinal fasciculus (SLF)

In both the 5-and 7-year-olds, our results showed that the left SLF did not relate to phonological awareness or reading skill, a pattern consistent with work suggesting that the AF is more important than the SLF for these skills in children (Yeatman et al., 2011, Saygin et al., 2013). Consistent with this, Saygin et al. (2013) reported correlations of phonological awareness with AF in 5–6-year-olds, but no comparable effects in ILF or SLF. However, there is also evidence that SLF is related to phonology. Dodson et al. (2018) noted a trend for a correlation of phonology to SLF in 6-year-olds, and Travis et al. (2017) found a significant association. A likely source of discrepancy is the different methods for measuring phonological awareness. Our analyses focused on CTOPP-2 Elision, whereas Dodson et al. (2018) and Travis et al. (2017) used broader CTOPP composites that included Elision, Sound Matching and Blending. Saygin et al. (2013) combined Elision, Blending, and Nonword Repetition. These differences in how phonological awareness is measured likely influences the nature of the brain-behavior correlations, so future work needs to determine whether these relations are specific to component skills such as the ability to segment, blend and manipulate phonemes.

### 5.3 Inferior fronto-occipital fasciculus (IFOF)

Our findings showed that the left IFOF was not related to semantic processing at age 5 but was uniquely associated with this skill at age 7, indicating emerging specialization in this ventral tract within this developmental window. The brain-behavior correlation was observed on a semantic similarity task to spoken words that did not involve reading (i.e. CELF-5, Word Classes). The selectivity of this result to oral language is consistent with accounts that link IFOF to semantic–lexical processing in non-reading contexts. Lesion and patient studies implicate left IFOF in semantic access (Han et al., 2013) and relate reduced ventral tract integrity, particularly IFOF, to semantic deficits (Surbeck et al., 2020). Models of language production posit a role of IFOF in semantic-to-lexical mapping (Shekari and Nozari, 2023).

Although we did observe correlations of IFOF to semantic processing, it was not related to reading skill. Our lack of finding with reading skill differs from other reports of IFOF being related to reading ability in younger children (Vanderauwera et al., 2017). It also differs from findings that report IFOF integrity relates to reading comprehension in poor decoders (Arrington et al., 2017). A likely explanation is the nature of the measure of reading skill. Our measure of reading skill was Letter–Word Identification that primarily taps in decoding and places less emphasis on semantic access. In contrast, study (Vanderauwera et al., 2018) showing links between IFOF and reading skill employed tasks that invite lexical strategies – e.g. children see three phonologically similar but orthographically different words and select the correctly spelled form. This task emphasizes the lexical route by requiring access to stored orthographic representations, morphological knowledge, and visual word familiarity to choose the correctly spelled form. Thus, the relation of IFOF to reading skill may be more evident on semantically demanding measures.

We found that the correlation between semantic skill and the IFOF was localized to nodes in the middle of the tract. The posterior IFOF contains divergent crossings with ILF (Alves-Belo, 2022), and AFQ studies often focus on the middle 80 % of IFOF to mitigate the influence of posterior crossing fibers (Huber et al., 2018, Vandecruys et al., 2024, Yeatman and Huber, 2019). Although we did not adopt a restriction, our brain-behavior correlation for mid-tract nodes is consistent this portion having greater directional coherence.

### 5.4 Inferior longitudinal fasciculus (ILF)

In our study, the left ILF was associated with reading skill in 7-year-olds, but not 5-year-olds. The ILF was not related to semantic skill in either group. This pattern suggests that ILF contributions are specific to reading and emerge later in development. The association of the ILF with reading skill aligns with previous literature. Other studies have reported that the ILF is correlated with early literacy skills in 5–8-year-olds (Broce et al., 2019) and relates to text comprehension in children of age 7–9. The ILF also relates to word reading/comprehension in adolescents (Horowitz-Kraus et al., 2014, 2015) and shows associations with reading in adolescents and adults (Yeatman et al., 2012, Grotheer et al., 2021). Together, these findings support a strengthening role for ILF in reading skill. Interestingly, we found that the relation of reading skill to the ILF at age 7 was negative. This is consistent with longitudinal evidence in this age that FA in the ILF declines over time in poorer readers but increases in better readers (Yeatman et al., 2012). The authors proposed a dual-process account in which myelination increases FA and axonal pruning decreases FA. This model suggests that synchrony of these processes yields lower FA in stronger readers in younger children followed by developmental increases, whereas asynchrony yields higher FA in weaker readers in younger children followed by developmental decreases. Our negative correlation at age 7, which is absent at age 5, is consistent with this developmental inflection point and implies non-linear ILF maturation relevant to reading outcomes.

**Limitations**

This study has some limitations that must be acknowledged. We recognize that there are differences in the standard scores, the 5-year-olds tended to score higher than the 7-year-olds on the language and reading measures, indicating a cohort effect. For the Letter-Word identification test this may be due to qualitative differences as the items for five-year-olds mostly consists of naming letters and reading simple words and those for the seven-year-olds consists of reading mostly longer, multisyllabic words (see also the supplementary information). Given the complexity of developmental changes and the multitude of factors influencing language and reading skills, future research should explore these questions by eliminating cohort differences. This approach would enhance the robustness of the findings and clarify the extent to which cohort effects or sample differences could impact the observed outcomes. It is to note that the differences between 5-year olds and 7-year-olds were relatively modest at less than one third of the standard deviation for the measures used in the brain-behavior analysis (Elision and Word Classes) to examine unique relations with phonology and semantics. Thus, overall, while this analysis has yielded valuable insights, the identified limitations underscore the necessity for careful interpretation of our findings.

## 6 Conclusion

The current results provide evidence for early specialization of the left AF for phonological processing only in 5-year-olds. This specialization overlapped with relationships between FA of the left AF and reading ability. This is consistent with empirical research and theoretical models which argue for the early importance of phonological processing to reading acquisition (Harm and Seidenberg, 1999, Harm and Seidenberg, 2004). We also showed later specialization of the left IFOF for semantic processing only in 7-year-olds, which did not show any correlations with reading ability. The lack of correlations with reading may be due to the nature of our reading skill measures which emphasized oral decoding. Overall, the progression of earlier specialization for phonological compared to semantic processing is consistent with neurocognitive models of language acquisition. Finally, the current results show a relationship between FA of the left ILF and reading ability only in 7-year-olds. Although this is overall consistent with studies emphasizing the importance of this tract in reading, the direction of the relationship suggests that there may be a non-linear relation depending on age.

## Funding

This research was supported by a 10.13039/100000002National Institutes of Health grant (R01 DC013274) and 10.13039/100000001National Science Foundation (BCS 1358794) grant to James R. Booth.

## CRediT authorship contribution statement

**Avantika Mathur:** Writing – review & editing, Writing – original draft, Visualization, Methodology, Investigation, Formal analysis, Data curation. **Huijia Zheng:** Writing – original draft, Formal analysis. **Yingying Wang:** Writing – review & editing, Software, Conceptualization. **Marjolein Mues:** Writing – review & editing. **James R. Booth:** Writing – review & editing, Supervision, Resources, Funding acquisition, Conceptualization.

## Declaration of Competing Interest

The authors declare no conflict of interest.

## Data Availability

The data is shared.
