# Supplementary material for: The association of dorsal and ventral white matter tracts with phonological and semantic processing of language in 5- to 7-year-old children
Source: Dev Cogn Neurosci. 2025 Dec 19;77:101662. doi: 10.1016/j.dcn.2025.101662 (PMC12807815; doi:10.1016/j.dcn.2025.101662)
Supplement: Supplementary file 1 — Supplementary material [file mmc1.docx]

# Supplementary Figures

Figure S1

*AFQ Tract profile, Superior Longitudinal Fasciculus (SLF)*


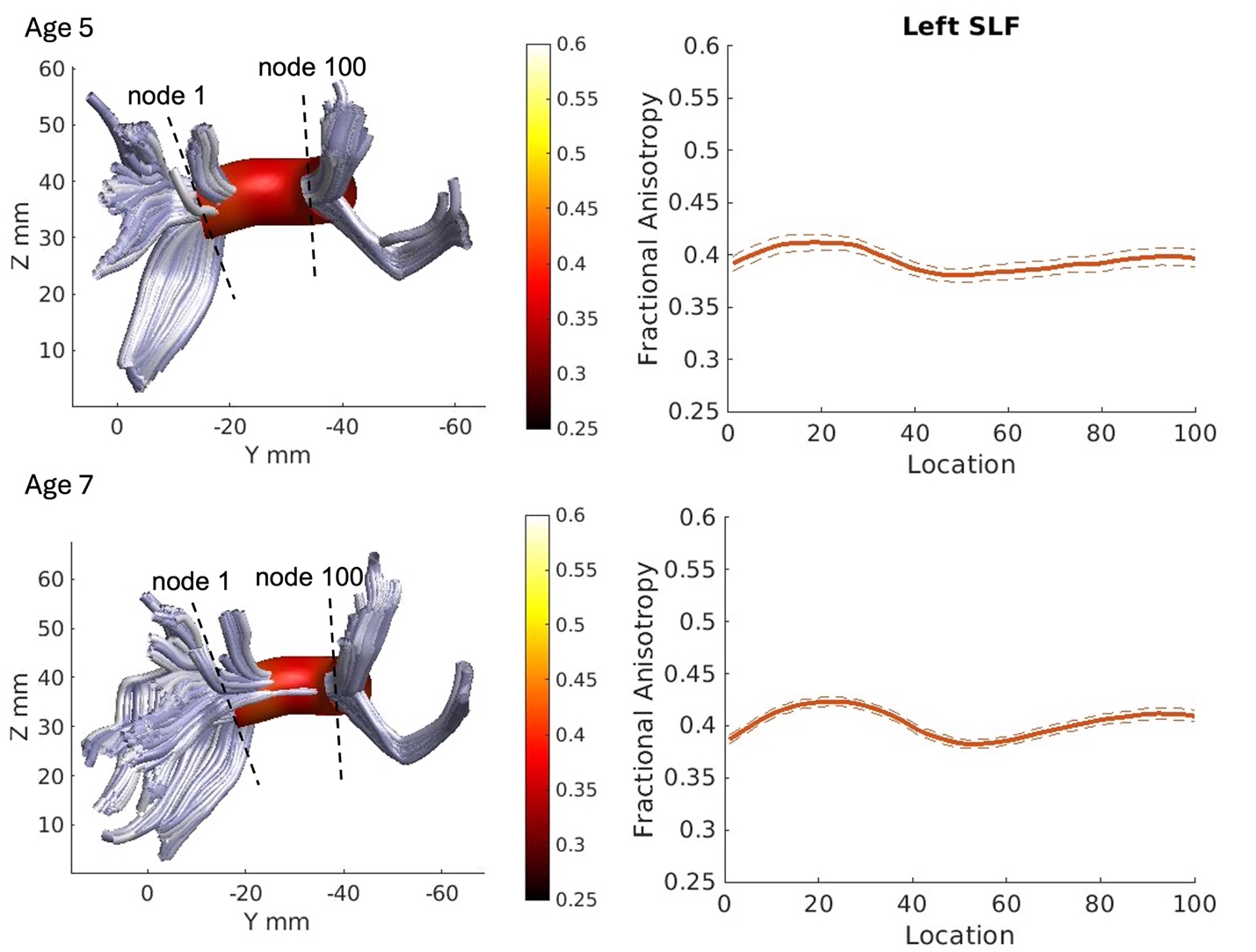


Note. Color bars show FA values; nodes 1 and 100 from AFQ are marked on tract profile.

Figure S2

*AFQ Tract profile, Arcuate Fasciculus (AF)*


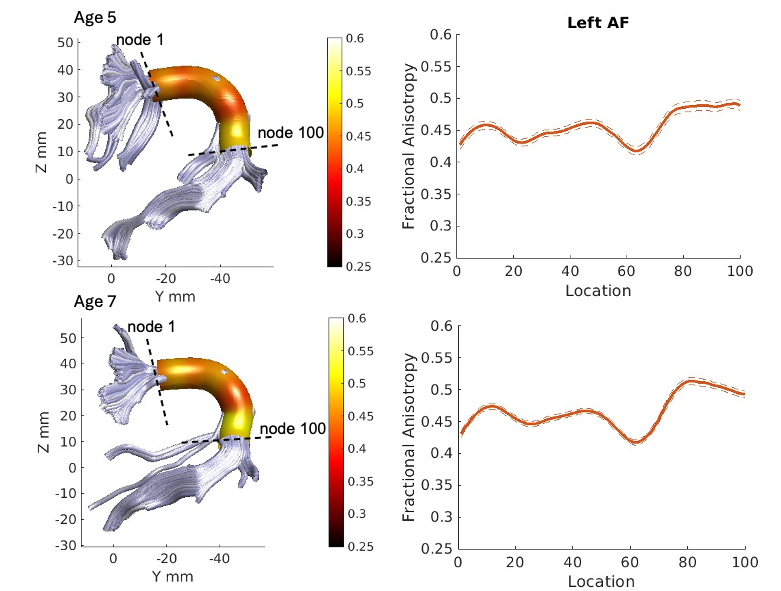


Note. Color bars show FA values; nodes 1 and 100 from AFQ are marked on tract profile.

Figure S3

*AFQ Tract profile, Inferior Longitudinal Fasciculus (ILF)*

Note. Color bars show FA values; nodes 1 and 100 from AFQ are marked on tract profile.

**
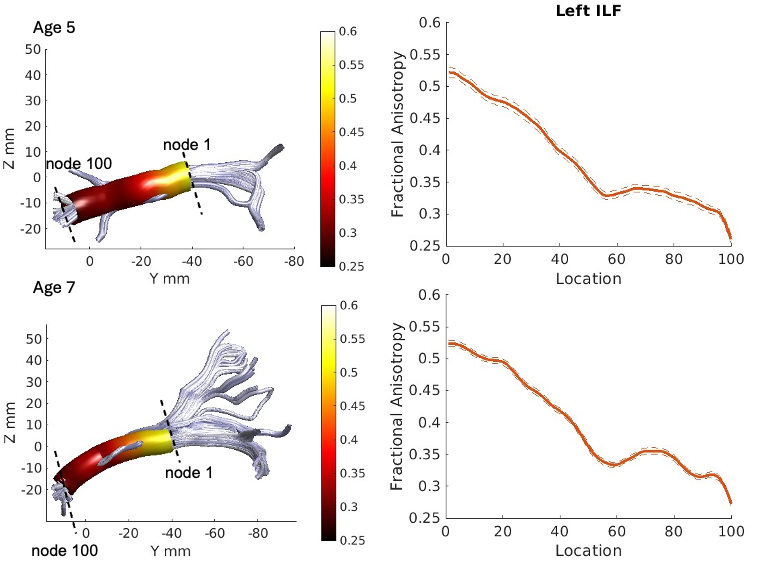
**

Figure S4

*AFQ Tract profile, Inferior Fronto-Occipital Fasciculus (IFOF)*
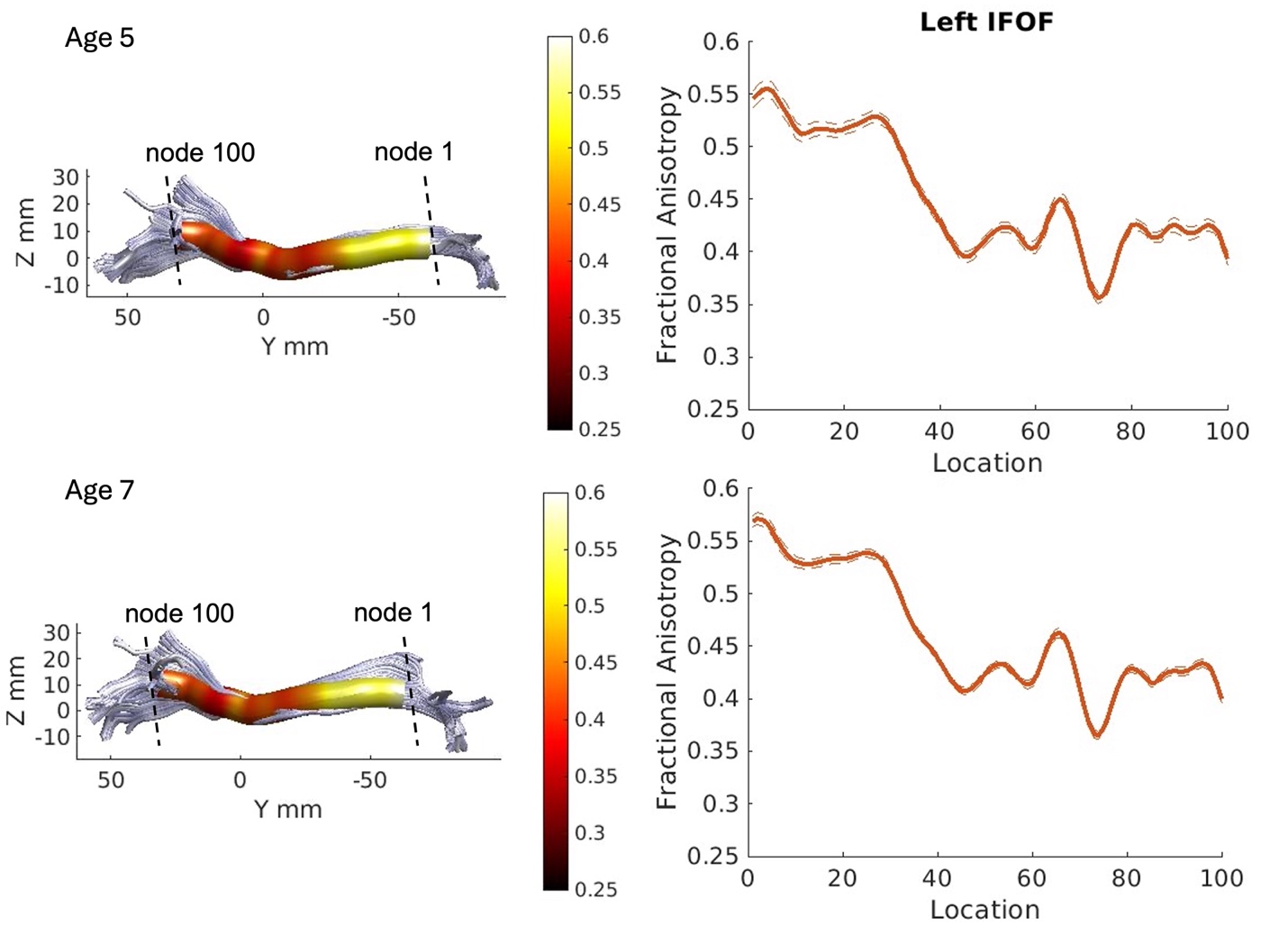


Note. Color bars show FA values; nodes 1 and 100 from AFQ are marked on tract profile.

**Figure S5**

White matter partial correlations of fractional anisotropy (FA) in the left arcuate fasciculus (AF) with behavioral measures in 5-year-olds for tracts resampled to 30 nodes

**
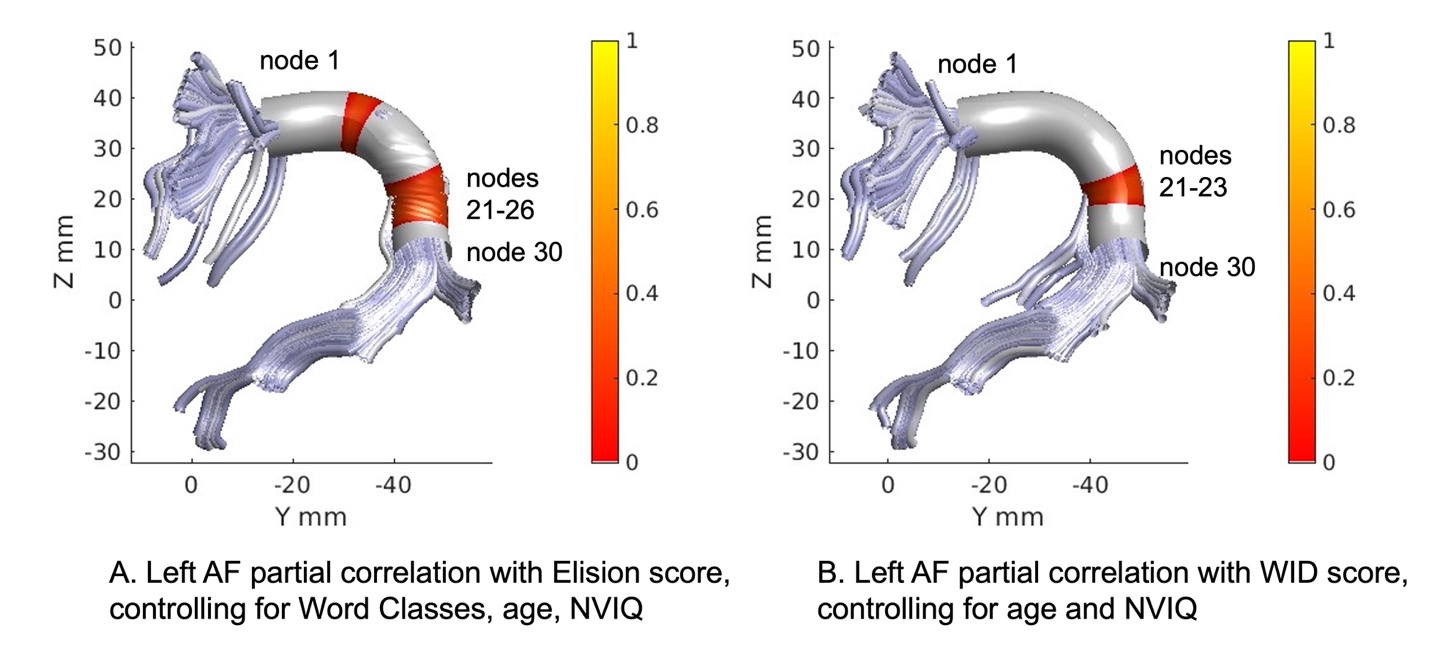
**

Note. (A) Positive partial correlation with phonological processing (Elision), while controlling for semantics (Word Classes), age and nonverbal intelligence (NVIQ). (B) Positive partial correlation with Letter-Word Identification (WID) while controlling for age and nonverbal intelligence (NVIQ) for tracts resampled to 30 nodes. The color bars show significant correlation values.

**Figure S6**

White matter partial correlations of fractional anisotropy (FA) in the left inferior fronto-occipital fasciculus (IFOF) and the left inferior longitudinal fasciculus (ILF) with behavioral measures in 7-year-olds for tracts resampled to 30 nodes

**
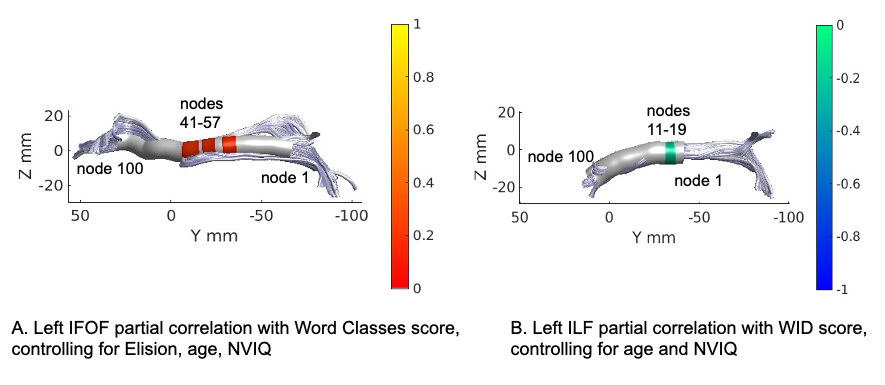
**

Note. (A) Positive correlation of the left inferior fronto-occipital fasciculus (IFOF) with semantics (Word Classes), while controlling for Elision, age and nonverbal intelligence (NVIQ). (B) Negative correlation of the left inferior longitudinal fasciculus (ILF) with Letter-Word Identification (WID) while controlling for age and nonverbal intelligence (NVIQ) for tracts resampled to 30 nodes. The color bars show significant r-values.

**Supplementary Material**

**Methods**

**Histograms of raw scores of Letter-Word identification test for 5 and 7 year olds**

Letter-Word identification measures the subject’s word identification skills. The initial items require the individual to identify letters that appear in large type, and the remaining items require the person to pronounce words correctly. The individual is not required to know the meaning of any word. The items become increasingly difficult as the selected words appear less frequently in written English. To best represent this score across the two cohorts, histogram plots of Letter-Word identification raw scores are plotted (see Figure S7). The plots in Figure S7 represent the Letter-Word identification raw scores for 5-year-olds (A) and 7-year-olds (B). A partially overlapping samples t-test (equal-variance) conducted to compare the standardized scores across the 5-year-old and 7-year-old cohort indicated that the 5-year-old cohort has a significantly higher average standardized Letter-Word Identification score than the 7-year-old cohort, (t (169.90) = 7.04, p < 0.001) (refer to Table 1). However, the raw scores show that the 5-year-olds named fewer words correctly than the 7-year-olds, with 5-year-olds mostly identifying letters and reading 2-5 letter words, whereas 7 years olds mostly reading longer words (see Figure S7)

**Figure S7** Letter-Word identification raw scores for 5-year-olds (A) and 7-year-olds (B).


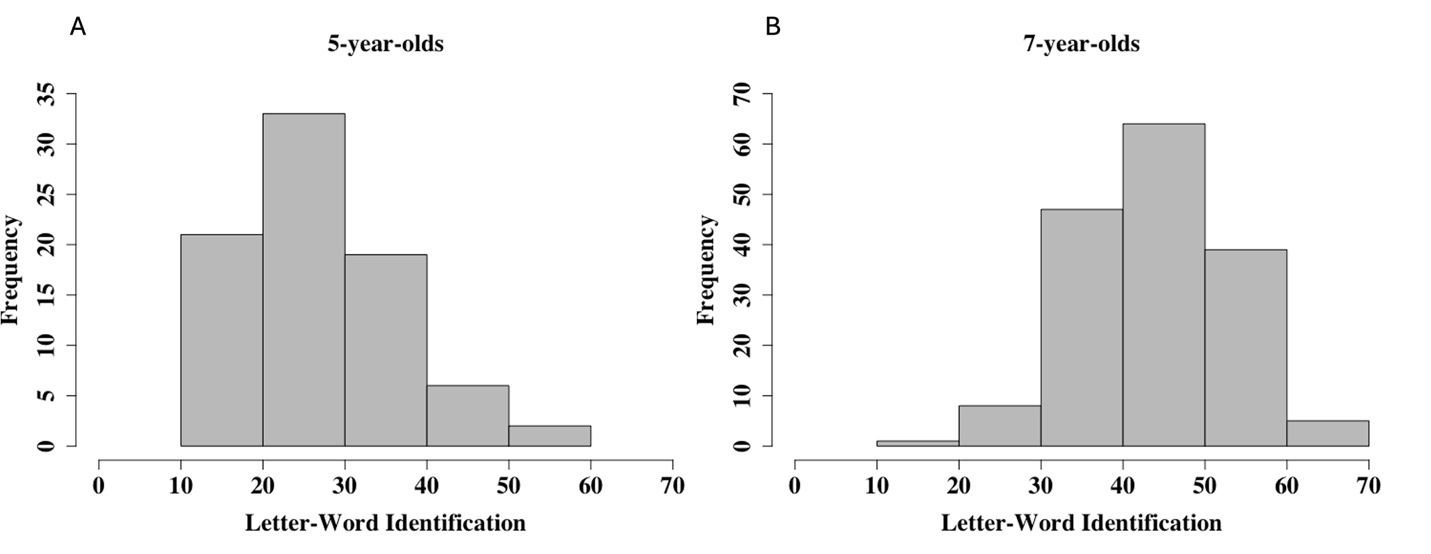


Note. The following table lists the letters to words of increasing complexity for each bin of raw scores on x-axis.

1-10 P E B C k r A D G cat

11-20 m h t b car on to dog in can

21-30 as get was have they when there must about only

31-40 part cloud because knew own whole against sentence island decide

41-50 since distance usually scientist bounties process experience moustache achieved tremenous

51-60 systematic urged ancient obviously sufficient particularly domesticated interpretation therapeutic bouquet

61-70 significance provincial aeronautic conspicuous diacritical deficiencies pituitary trivialities debutante magnanimous

71-76 homogenization indissolubly picaresque ubiquitous argot satiate

**Histograms of raw scores of Elision test for 5 and 7 year olds**

Phonological processing was evaluated with the Elision subtest from the Comprehensive Test of Phonological Processing, 2nd Edition (CTOPP-2; (Wagner et al., 2013). This subtest asks participants to remove a phoneme from a spoken word to form a new spoken word, for instance, saying “tiger” without the “g” would result in “tire”. A partially overlapping samples t-test (equal-variance) conducted to compare the scaled scores across the 5-year-old and 7-year-old cohort indicated that the 5-year-old cohort has a significantly higher average scaled Elision score than the 7-year-old cohort, (t (142.79) = 2.01, p = 0.05) (refer to Table 1). However, the histogram plot shows that the raw scores of most of the 7-year-olds were higher than the 5-year-olds (see Figure S8).

**Figure S8**

**
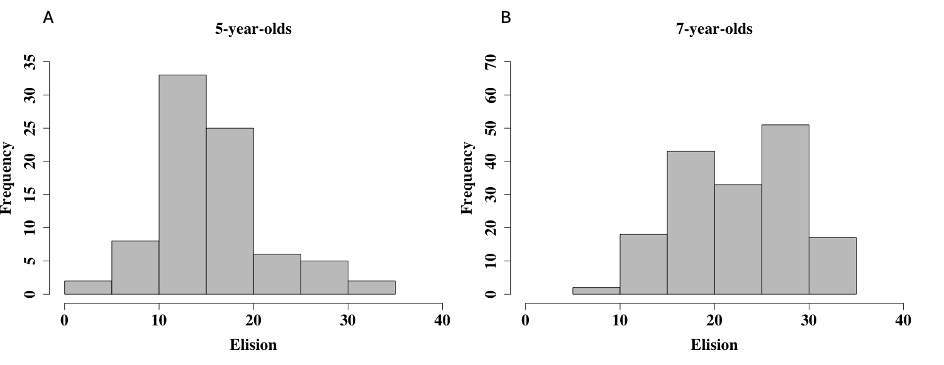
**

Note. The plots in Figure S8 represent the Elision raw scores for 5-year-olds (A) and 7-year-olds (B)

**Histograms of raw scores of Word Classes subtest for 5 and 7 year olds**

Semantic processing was assessed with the Word Classes subtest (CELF-5; Wiig et al., 2003). This subtest instructs participants to choose two words with a semantic relationship from three or four presented words, for instance, choose “puppy” and “dog” when presented with “puppy, dog, frog”. A partially overlapping samples t-test (equal-variance) conducted to compare the scaled scores across the 5-year-old and 7-year-old cohort indicated that the 5-year-old cohort has a significantly higher average scaled Word Classes score than the 7-year-old cohort, (t (169.90) = 2.06, p = 0.04) (refer to Table 1). However, the histogram plot shows that the raw scores of most of the 7-year-olds were higher than the 5-year-olds (see Figure S9)

**Figure S9**

**
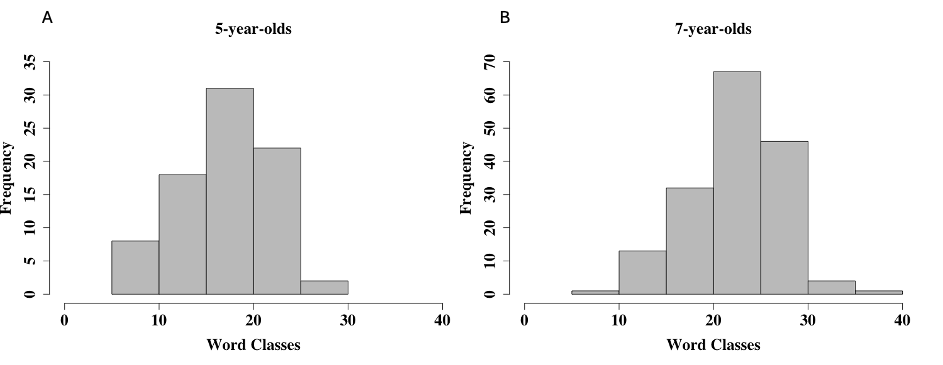
**

Note. The plots in Figure S9 represent the Word Classes raw scores for 5-year-olds (A) and 7-year-olds (B)

**Results**

**Results obtained from resampling the tracts to 30 nodes**

In 5-year-olds, resampling the tracts to 30 nodes yielded the same results, the left AF (nodes 11-12, 21-26) showed a positive partial correlation with the Elision score, with the second cluster between nodes 21-26 surviving the stringent threshold of FWE cluster size correction for adjacent nodes ≥ 5, at p < 0.05 uncorrected. The overlapping left AF (nodes 21-23) showed a positive partial correlation with Word Identification scores (the cluster survived the lenient threshold for adjacent nodes ≥ 3 at p < 0.05 uncorrected) (refer to supplementary Figure S5). The non-significant results remained the same after resampling, such that no significant correlations passing the cluster correction were observed between FA of SLF and the Elision score or the Word Identification score in 5-year-olds. No significant correlations passing the cluster correction were observed between FA of ventral tracts and the Word Classes score or the Word Identification score in 5-year-olds.

In 7-year-olds, resampling the tracts to 30 nodes yielded the same results, the left IFOF (nodes 10-11,13-14,16-17), showed a positive correlation with Word Classes scores at p < 0.05 uncorrected. While the nodes were close together, they did not survive the lenient threshold for adjacent nodes ≥ 3. The left ILF (nodes 4-6) showed a negative correlation with the Word Identification score at the lenient threshold for adjacent nodes ≥ 3 at p < 0.05 uncorrected (refer to supplementary Figure S6). The non-significant results remained the same after resampling such that no significant correlations passing the cluster correction were observed between FA of dorsal tracts and the Elision score or the Word Identification score in 7-year-olds.

**Results for partial correlations obtained without controlling for the other process**

Based on the reviewer’s suggestion for comparison with other published studies, we conducted partial correlations for each of the tracts and groups (age 5 and age 7) for phonological processing and semantic processing without controlling for the other process (only controlling for age and NVIQ) for broader comparison to other studies. To find nodes within the dorsal tracts (left AF and left SLF) that were related to phonological processing, we performed a partial correlation analysis between Elision score and the FA value at each node along the tracts, controlling for age and NVIQ. To find nodes within the ventral tracts (left IFOF and left ILF) that were related to semantic processing, we performed a partial correlation analysis between Word Classes score and the FA value at each node along the tracts, controlling age and NVIQ.

In 5-year-olds, a partial correlation analysis revealed a positive correlation between FA of left AF and the Elision score (nodes 33-39, 78-87, both at p < 0.05 uncorrected). Another partial correlation analysis revealed a positive correlation between FA of left SLF and the Elision score (nodes 65-79, p < 0.05 uncorrected). No significant correlations were observed between FA of ventral tracts and the Word Classes score.

In 7-year-olds, a partial correlation analysis revealed a positive correlation between FA of left IFOF and the Word Classes score (nodes 41-46, 50-57, both at p < 0.05 uncorrected). The FA of left ILF showed no significant correlation with the Word Classes score. No significant correlations were observed between FA of dorsal tracts and the Elision score.

**Results for partial correlations obtained for 45 participants overlapping across age 5 and age 7**

Based on the reviewer’s suggestion, we conducted analyses on smaller sample that had complete data at both time points. In 5-year-olds, a partial correlation analysis revealed no significant positive correlation between FA of dorsal tracts (i.e. left AF and left SLF) and the Elision score or between FA of ventral tracts (i.e. left IFOF and left ILF) and the Word Classes score. In 7-year-olds, a partial correlation analysis revealed no significant positive correlation between FA of ventral tracts and the Word Classes score or between FA of dorsal tracts and the Elision score.

We have incorporated the partial correlation heat maps for FA of left AF with phonological processing (Elision), while controlling for semantics (Word Classes), age and nonverbal intelligence (NVIQ) in 5-year-olds (n=45) (Figure S10A) and FA of left IFOF with semantics (Word Classes), while controlling for Elision, age and nonverbal intelligence in 7-year-olds (n=45) (Figure S10B). The results are consistent with our main results with the larger sample as correlations remain positive between left AF FA and Elision in 5-year-olds and between left IFOF FA and Word classes in 7-year-olds, but effect sizes are diminished and does survive statistical significance given the reduction in sample size of overlapping participants.

**Figure S10**


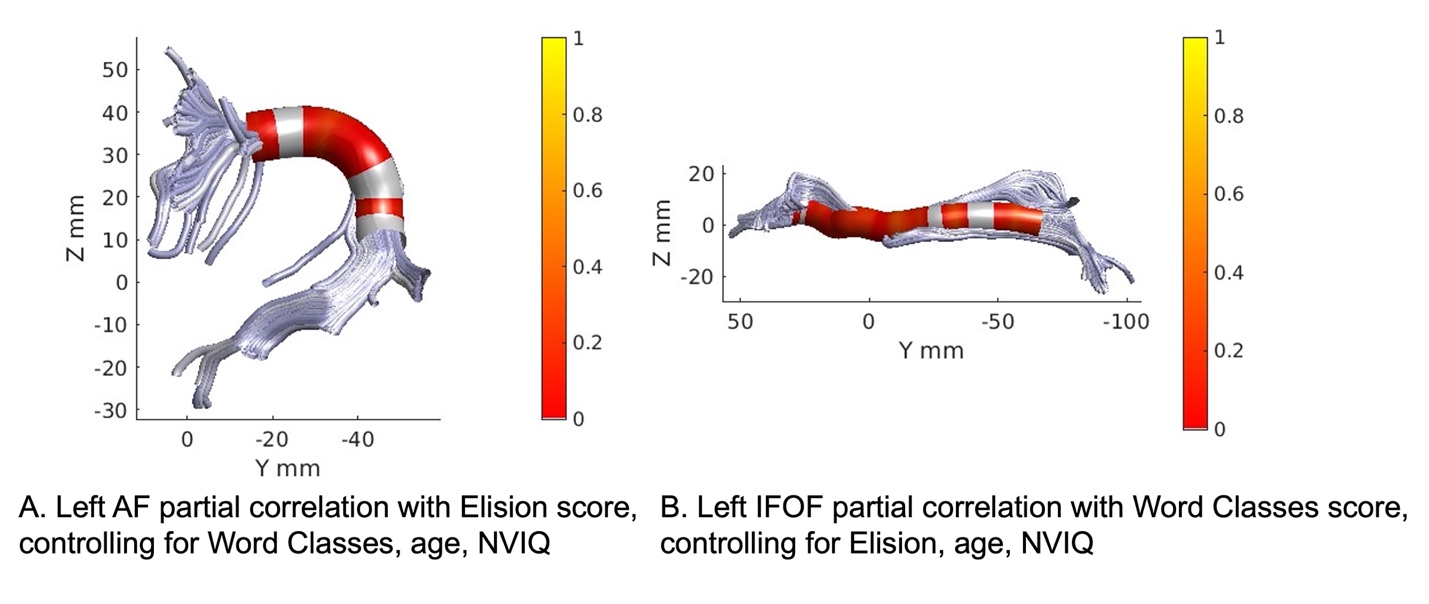


Note. (A) Positive partial correlation with phonological processing (Elision), while controlling for semantics (Word Classes), age and nonverbal intelligence (NVIQ) in 5-year-olds (n=45). (B) Positive partial correlation of the left inferior fronto-occipital fasciculus (IFOF) with semantics (Word Classes), while controlling for Elision, age and nonverbal intelligence (NVIQ) in 7-year-olds (n=45).
